# BTG2 suppresses renal cell carcinoma progression through N6-methyladenosine

**DOI:** 10.3389/fonc.2022.1049928

**Published:** 2022-12-14

**Authors:** Fuming Qi, Wenlong Liu, Bo Tan, Juan Zhang, Yan Ma, Congcong Cao, Fujun Ma, Bo Zhu, Jinhui Yang, Xiaoyun Liu

**Affiliations:** Urology Department, Shengli OilField Central Hospital, Dongying, Shandong, China

**Keywords:** BTG2, m^6^A, RCC, dCas13b, Mettl3

## Abstract

The biological functions of N6-methyladenosine (m^6^A) modification of mRNA have recently received a great deal of attention. In previous studies, m^6^A methylation modification has been shown to regulate mRNA fate and to be crucial for the progression and development of tumors. BTG2 (B-cell translocation gene 2) is a member of BTG/TOB anti-proliferative protein family. BTG2 could inhibit cell proliferation and migration and regulate the cell cycle progression. In this study, we confirm that BTG2 is frequently down-regulated in renal cell carcinoma (RCC) tissues and its low expression is associated with unfavorable prognosis and decreased m^6^A level. Moreover, we found that m^6^A methylation modifies the 5’UTR of BTG2 to promote its mRNA stability by binding to IGF2BP2. It has been shown that CRISPR/dCas13b-METLL3 can specifically increase BTG2 m^6^A modification to significantly increase its m^6^A and expression levels. Then m^6^A hypermethylation in BTG2 mRNA could dramatically inhibit RCC cells proliferation and migration, and induce cells apoptosis. Taken together, our data show that BTG2 functions as a tumor suppressor and is frequently silenced *via* m^6^A modification in RCC.

## Introduction

Renal cell carcinoma (RCC) is one of the common malignant tumors of the urinary system, and its incidence is 5% in men and 3% in women, and its incidence is gradually increasing (1, 2). Its etiology is not clear, and its pathogenesis may be closely related to genetic factors, smoking, obesity, diet, hypertension and so on. Genetic mutations are an important cause of RCC and can be detected early (3). With the development of gene therapy and the in-depth study on the mechanism of various tumor suppressor genes, it brings new directions and ideas for the treatment of tumors to find targets from gene level.

RNA modifications play an essential role in regulating gene expression post-transcriptionally. Eukaryotes have the most abundant internal modification, m^6^A, which constitutes 0.1-0.3% of total adenosine residues (4, 5). It is located in the non-coding 3’term, near the stop codon and long internal exons, and is related to RNA stability, splicing, intracellular distribution, and translation (6, 7). The cellular m^6^A state is controlled by a group of genes called “writers” (WTAP, METTL3 and METTLL4), “erasers” (FTO and ALKBH5), and “readers” (YTHDF1/2/3, IGF2BP2/3, YTHDCL and YTHDC2) (8–13). N6-methyladenosine RNA modifications on the sixth nitrogen atom of adenine, one of the most important nitrogen atoms in RNA, have become one of the hottest topics in various human diseases including hypertension (14), cardiac hypertrophy (15), viral infection (16), diabetes (17) and cancers (18, 19). Yet, the expression patterns of RNA m^6^A methylation modification and their underlying mechanisms remain largely unknown in RCC.

B-cell translocation gene 2 (BTG2) belongs to the anti-proliferation gene family and is an early growth response gene. Many studies have shown that BTG2 participates in the regulation of cell growth, differentiation and apoptosis (20, 21). At the same time, the expression of BTG2 in many types of cancer cells has varying degrees of reduction or even direct deletion, while the expression level of BTG2 gene in many corresponding normal cells is relatively higher. This suggests that abnormal expression of BTG2 is closely related to tumor formation, suggesting that tumor formation may be related to BTG2 expression (22–24). BTG2 expression level has been found to be correlated with the clinical characteristics of the tumor in breast cancer samples. The lower the level of BTG2 expression, the higher the risk of lymphatic invasion and vascular invasion in breast cancer patients, and the higher the risk of tumor metastasis. Low expression of BTG2 is associated with tumor recurrence. And low expression of BTG2 is associated with high tumor recurrence rate and low overall survival rate (25). However, the role of BTG2 in the growth and migration of RCC remains unclear.

In this study, we observed RCC tissues exhibited significantly reduced BTG2 m^6^A level and expression level relative to normal tissues. Preliminary experimental results showed that methylation of 5’UTR in BTG2 mRNA can regulate its mRNA stability by recruiting m^6^A reader protein IGF2BP2. Further, upregulation of BTG2 m^6^A methylation level can inhibit RCC cells proliferation and migration, and promotes RCC cells apoptosis by dCas13b-METTL3.

## Results

### Downregulation of BTG2 is associated with unfavourable prognosis and decreased m^6^A methylation modification levels in RCC

**Figure 1:**
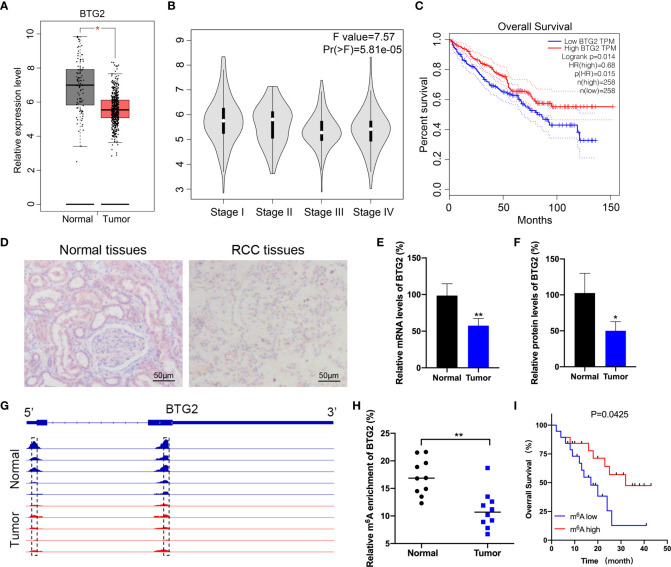
BTG2 expression and m^6^A methylation level in RCC patients. **(A)** An analysis of BTG2 mRNA expression in RCC tumor tissues (red plot) and normal tissues (grey plot) is shown in the box plot below based on TCGA database. **(B)** The expression levels of BTG2 in different stages of RCC based on TCGA database. **(C)** The survival plot for RCC patients stratified by expression of BTG2. **(D)** The protein expression levels of BTG2 in tumor tissues and normal tissues analyzed by IHC. **(E)** The mRNA expression levels of BTG2 in tumor tissues and normal tissues analyzed by RT-qPCR. **(F)** The protein expression levels of BTG2 in tumor tissues and normal tissues analyzed by western blot. **(G)** The m^6^A peaks within BTG2 mRNA in tumor tissues and normal tissues analyzed by IGV. **(H)** The m^6^A enrichment of BTG2 in tumor tissues and normal tissues analyzed by MeRIP-qPCR. **(I)** The survival plot for RCC patients stratified by m^6^A level of BTG2. * P<0.05, ** P<0.01.

BTG2 has been found to be expressed at low levels in a variety of cancer, which has potential role in the development and subsequent progression of tumors. However, there is limited evidence regarding the regulatory pathways that control the expression levels of BTG2 in RCC. According to The Cancer Genome Atlas (TCGA) database, we first found that BTG2 expression levels are downregulated in RCC tissues compared with adjacent normal tissues (**Figures 1A, B**). We then examined the impact of BTG2 expression on patient survival using TCGA dataset. The results showed that there was a significantly negative correlation between low expression of BTG2 and poor survival rates in patients (**Figure 1C**). In addition, we also performed IHC and RT-qPCR experiments on RCC and normal tissues to determine the expression levels of BTG2 protein and mRNA. The results showed significant downregulation of BTG2 in tumor tissues compared to adjacent normal tissues (**Figures 1D–F**). The expression levels of METTL3 and IGF2BP2 were also significantly decreased in RCC tumor tissues compared to normal tissues (**Supplementary Figure S1**).

It is evident that m^6^A RNA methylation plays an important role in tumorigenesis by inhibiting tumor suppressor genes. Based on previous MeRIP-seq results, we examined the m^6^A methylation level of BTG2 in RCC tissues to investigate the role of m^6^A methylation modification in RCC progression. The data revealed that the m^6^A peaks on BTG2 mRNA were located in its 5′UTR and CDS region. And compared to the normal tissue, tumor tissues showed lower peak enrichment (**Figure 1G**). MeRIP-qPCR results validated that the m^6^A methylation level of BTG2 was significantly decreased in tumor tissues compared to normal tissues (**Figure 1H**). Based on the Kaplan-Meier survival analysis, RCC patients with lower m^6^A levels of BTG2 had a shorter overall survival (OS) (**Figure 1I**). Overall, BTG2 expression and m^6^A methylation levels were correlated with overall survival of RCC patients.

### BTG2 mRNA stability is regulated by m^6^A modification in RCC cells

**Figure 2:**
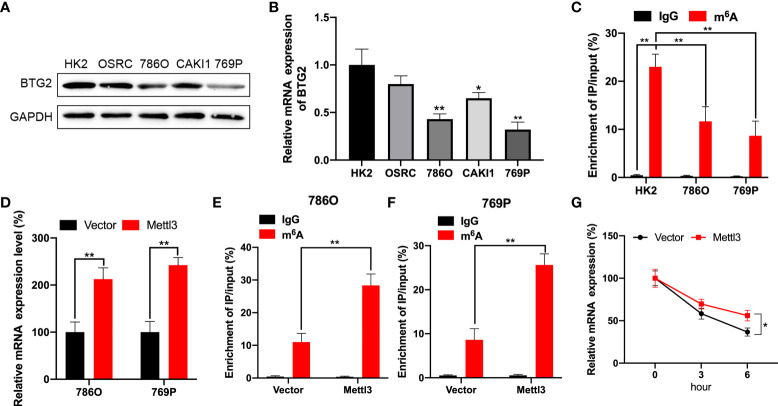
BTG2 expression and m^6^A methylation level in RCC cells. **(A)** The protein expression levels of BTG2 in RCC cells were detected using western blot. **(B)** The mRNA expression levels of BTG2 in RCC cells were detected using RT-qPCR. **(C)** The m^6^A methylation levels of BTG2 in RCC cells were detected using MeRIP-qPCR. **(D)** The mRNA expression levels of BTG2 in 786O and 769P cells after overexpressing Mettl3 were detected using RT-qPCR. E-F. The m^6^A methylation levels of BTG2 in 786O cell **(E)** and 769P cells **(F)** after overexpressing Mettl3 were detected using MeRIP-qPCR. **(G)** The mRNA expression levels of BTG2 in 786O cells after overexpressing Mettl3 and treatment with ActD for indicated times were detected using RT-qPCR. * P<0.05, ** P<0.01.

Using Western blot and RT-qPCR, we first detected BTG2 expression in RCC cells to explore possible mechanisms involved in m^6^A regulation of BTG2 expression. As compared to normal renal tubule HK-2 cell line, BTG2 expression was significantly decreased in other RCC cell lines except for OSRC cell (**Figures 2A**, **2B** and **Supplementary Figure S2**). Further, based on MeRIP-qPCR results, BTG2 m^6^A enrichment in 786O and 769P RCC cells were significantly decreased in comparison with HK-2 cells (**Figure 2C**). Then we examined the mechanisms that influence BTG2 expression in RCC by the m^6^A methylation modification. As shown by RT-qPCR results, Mettl3-overexpressing 786O and 769P cells exhibited higher expression of BTG2 (**Figure 2D**). In addition, the m^6^A modification levels of BTG2 were also significantly increased in 786O and 769P cells with overexpression of Mettl3 (**Figures 2E, F**). To block transcription, we treated control cells and 786O cells overexpressing Mettl3 with Act-D. In RNA stability assays, Mettl3 overexpression prolonged BTG2 mRNA half-life (**Figure 2G**). Totally, our results showed that the m^6^A methylation modification in BTG2 mRNA might delay its degradation.

### BTG2 is regulated by m^6^A modification through methylation sites

**Figure 3:**
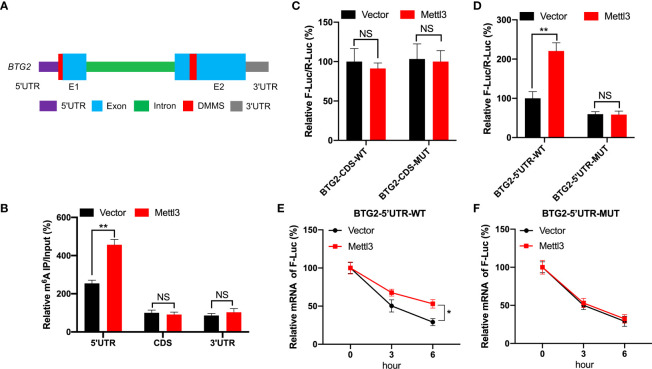
BTG2 is regulated by m^6^A modification through methylation sites. **(A)** Diagram showing DMMs positions within BTG2 mRNA. **(B)** The MeRIP-qPCR assay was used to analyze the m^6^A enrichment in different regions of BTG2 mRNA in control or Mettl3 overexpression 786O cells. **(C)** F-Luc/R-Luc activity in control and Mettl3-overexpressing 786O cells transfected with pmirGLO-BTG2-CDS-WT or pmirGLO-BTG2-CDS-MUT. **(D)** F-Luc/R-Luc activity in control and Mettl3-overexpressing 786O cells transfected with pmirGLO-BTG2-5’UTR-WT or pmirGLO-BTG2-5’UTR-MUT. E-F. The mRNA expression levels of pmirGLO-BTG2-5’UTR-WT **(E)** or pmirGLO-BTG2-5’UTR-MUT **(F)** in 786O cells after overexpressing Mettl3 and treatment with ActD for indicated times. * P<0.05, ** P<0.01, NS means No significance.

Based on MeRIP-seq data, there were two differentially methylated m^6^A peaks (DMMs) in BTG2 mRNA’s 5′UTR and CDS regions (**Figures 1G**, **3A**). Then, RNA fragments isolated from RCC cell are immunoprecipitated with anti- m^6^A antibody to characterize BTG2 mRNA methylation modification. According MeRIP-qPCR results, we found that the 5′UTR region in BTG2 mRNA had the highest m^6^A modification level, followed by the 3′UTR and CDS (**Figure 3B**). In addition, Mettl3-overexpressing cells showed increased enrichment of m^6^A in BTG2 5′UTR, which suggests that m^6^A modification in the 5′UTR is more dynamic than in the CDS region (**Figure 3B**). In order to investigate the possible roles of m^6^A methylation in the BTG2 mRNA CDS region, we used luciferase reporters containing the WT or MUT BTG2 CDS in RCC cells. The results showed that the luciferase activity of BTG2-CDS-MUT was similar between Mettl3-overexpressing and control groups compared to BTG2-CDS-WT (**Figure 3C**). In summary, these results indicate that m^6^A methylation level in the CDS region of BTG2 mRNA does not correlate with m^6^A modification, which promotes its mRNA stability.

Therefore, we tested whether BTG2 mRNA stability is regulated by m^6^A methylation modification in the 5′UTR region. In Mettl3-overexpressing RCC cells, BTG2-5′UTR-WT produced significantly higher luciferase activity than control group by the dual-luciferase assay (**Figure 3D**). While in Mettl3-overexpressing RCC cells, BTG2-5′UTR-MUT didn’t have a significant upregulation of luciferase activity than control group (**Figure 3D**). Moreover, the stability of BTG2-5′UTR-WT mRNA in Mettl3-overexpressing cells was increased over control cells (**Figure 3E**), while Mettl3-overexpressing cells and control cells didn’t have different mRNA half-lives of BTG2-5′UTR-MUT (**Figure 3F**). In conclusion, BTG2 mRNA stability is mediated by m^6^A methylation modifications in the mRNA 5′UTR region.

### IGF2BP2 involved in m^6^A-regulated expression of BTG2

**Figure 4:**
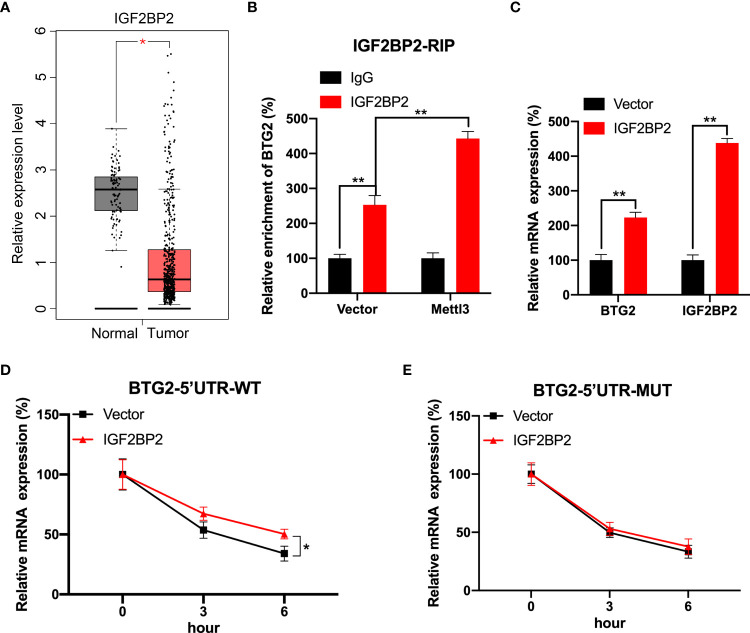
IGF2BP2 involved in m^6^A-regulated expression of BTG2. **(A)** An analysis of IGF2BP2 expression level in RCC tumor tissues (red plot) and normal tissues (grey plot) is shown in the box plot below based on TCGA database. **(B)** An analysis of BTG2 mRNA in control or Mettl3-overexpressing 786O cells using IGF2BP2 RIP-qPCR. **(C)** The expression levels of BTG2 and IGF2BP2 in control or IGF2BP2-overexpressing 786O cells using RT-qPCR. D-E. The mRNA expression levels of pmirGLO-BTG2-5’UTR-WT **(D)** or pmirGLO-BTG2-5’UTR-MUT **(E)** in 786O cells after overexpressing IGF2BP2 and treatment with ActD for indicated times. * P<0.05, ** P<0.01.

We further investigated mechanisms involved in m^6^A-regulated BTG2 mRNA stability. Several studies have shown that m^6^A modification can affect mRNA stability *via* readers such as IGF2BP1∼3 (26, 27). Based on the TCGA database, IGF2BP2 was evidently downregulated in RCC tumour samples compared to normal samples (**Figure 4A**). Consequently, we tested whether IGF2BP2 enhances BTG2 mRNA stability in a m^6^A-dependent manner. Through RIP-qPCR assays, we first detected that IGF2BP2 binds to BTG2 m^6^A modification sites. It was found that IGF2BP2 strongly binds to BTG2 mRNA in RCC cells (**Figure 4B**). Furthermore, compared to control cells, Mettl3-overexpressing cells had significantly higher binding between IGF2BP2 and BTG2 (**Figure 4B**). Then we overexpressed IGF2BP2 in RCC cells. It was found that IGF2BP2 could increase BTG2 mRNA levels (**Figure 4C**). Moreover, IGF2BP2 can increase mRNA stability in BTG2-5′UTR-WT (**Figure 4D**), but not in BTG2-5′UTR-MUT (**Figure 4E**). In our study, IGF2BP2 was proved to be implicated in m^6^A methylation-dependent BTG2 mRNA stabilization.

### CRISPR/dCas13b-METTL3 specifically targets m^6^A methylation of BTG2 to regulate its expression

**Figure 5:**
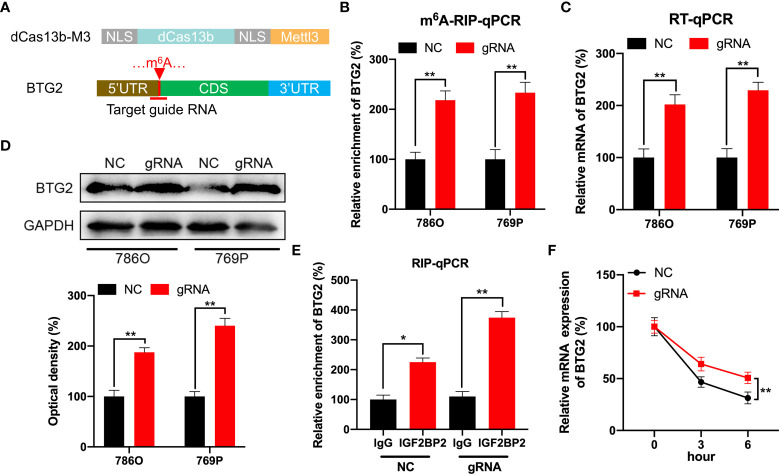
CRISPR/dCas13b-METTL3 specifically targets m^6^A methylation of BTG2 to regulate its expression. **(A)** Positions of m^6^A sites within BTG2 mRNA along with target guide RNA regions. **(B–D)**. The m^6^A **(B)**, mRNA **(C)** and protein levels **(D)** of BTG2 in RCC cells transfected with dCas13b-M3 combined with gRNA control or gRNA for BTG2, respectively. **(E)** A RIP-qPCR analysis of BTG2 mRNA is performed by using antibodies against IGF2BP2 on 786O cells transfected with dCas13b-M3 combined with gRNA control or gRNA for BTG2, respectively. **(F)** The mRNA expression levels of BTG2 in 786O cells transfected with dCas13b-M3 combined with gRNA control or gRNA for BTG2 and treatment with ActD for indicated times. * P<0.05, ** P<0.01.

In order to specifically methylate the m^6^A of BTG2, we combined a catalytically dead Cas13 enzyme with METTL3 (dCas13b-M3) (28). To target the BTG2 mRNA, guide RNAs were positioned around the m^6^A site in BTG2 mRNA (**Figure 5A**). As a first step, MeRIP-qPCR was performed on 786O and 769P cells to confirm that dCas13b-M3 induced m^6^A methylation of BTG2 (**Figure 5B**). Furthermore, we found that dCas13b-M3 targeting BTG2 significantly increased BTG2 mRNA and protein levels in 786O and 769P cells (**Figures 5C, D**). It may be due to dCas13b-M3 targets for BTG2 can significantly enhance BTG2 mRNA binding to IGF2BP2 (**Figure 5E**). Then the effects of dCas13b-M3 with control or gRNA for BTG2 mRNA were compared in order to determine whether m^6^A-mediated mRNA stability relates to dCas13b-M3-induced methylation upregulation. Based on the results of our study, targeted m^6^A methylation of BTG2 significantly lengthened its mRNA half-life, suggesting that dCas13b-M3 methylated m^6^A at 5′UTR region to increase its mRNA stability (**Figure 5F**).

### Specifical m^6^A methylation of BTG2 inhibits RCC cells proliferation and promotes cell apoptosis by CRISPR/dCas13b-METTL3

**Figure 6:**
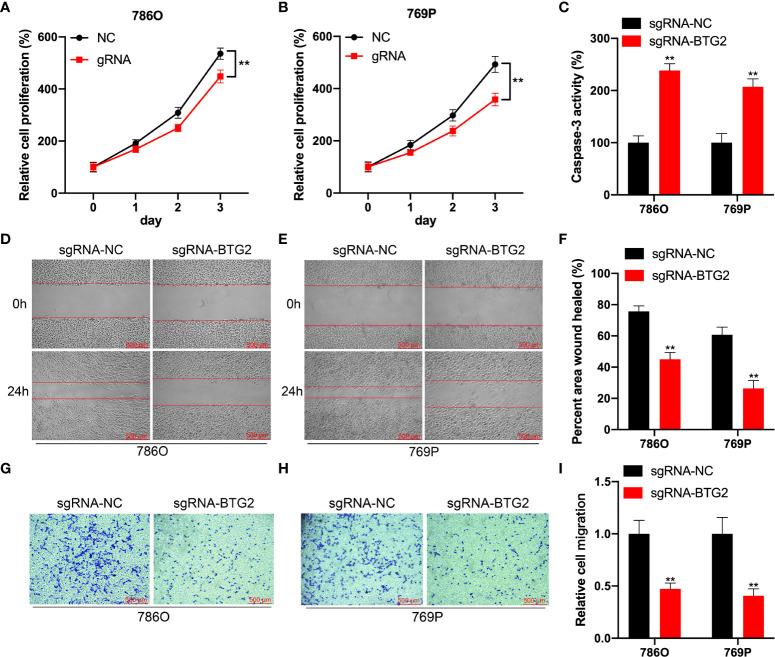
Specifical m^6^A methylation of BTG2 inhibits RCC cells proliferation and promotes cell apoptosis by CRISPR/dCas13b-METTL3. **(A, B)**. The cell proliferation of 786O **(A)** and 769P **(B)** cells transfected with dCas13b-M3 combined with gRNA control or gRNA for BTG2 was detected by CCK8 assay. **(C)** The cell apoptosis of 786O **(A)** and 769P **(B)** cells transfected with dCas13b-M3 combined with gRNA control or gRNA for BTG2. **(D-F)** Wound-healing assays for migration ability of 786O **(D)** and 769P **(E)** cells after transfected with dCas13b-M3 combined with gRNA control or gRNA for BTG2. **(G-I)**. Transwell assays for migration ability of 786O **(D)** and 769P **(E)** cells after transfected with dCas13b-M3 combined with gRNA control or gRNA for BTG2. ** P<0.01.

Further investigation to detect the role of BTG2 m^6^A hypermethylation might regulate RCC cell homeostasis was performed by detecting cell proliferation, migration and apoptosis in RCC cells. According to our results, BTG2 gRNA significantly decreased cell proliferation and promoted apoptosis in comparison with non-targeted control gRNA transfected with dCas13b-M3 in 786O and 769P cells (**Figures 6A–C**). According to wound healing assays and cell migration assays, hypermethylation of BTG2 inhibited cell migration in 786O and 769P cells (**Figures 6D–I**). These results showed that BTG2 could inhibit the RCC cells proliferation and migration, and promote RCC cells apoptosis through m^6^A methylation modification.

## Discussion

Renal cell carcinoma includes many histological subtypes with different biological manifestations and prognosis. The most basic histological subtypes include clear cell carcinoma of the kidney (75-80%), papillary carcinoma (10-15%), and chromocytoma (4-6%). Among them, renal clear cell carcinoma is the most common histological type, and is the most aggressive tumor subtype with the worst prognosis among all renal cell carcinoma subtypes. Surgical resection is the main treatment in clinical practice. For patients with advanced renal cancer who are not suitable for surgery, radiotherapy, chemotherapy and hormone therapy are the alternative treatment methods. However, clinical practice has proved that patients with renal cancer have low sensitivity to radiotherapy, chemotherapy and hormone therapy. Molecular targeted drug therapy is the main treatment for advanced renal cancer and recurrent renal cancer in recent years.

A reversible and dynamic RNA modification, m^6^A is a result of both a methyltransferase and demethylase protein. There is evidence that m^6^A modification plays a role in multiple cellular processes, such as heat shock (29), UV light (30), hypoxic stress (31), and oxidative stress (32). It has been reported that m^6^A modification is associated with the development of tumours, but their involvement in RCC is less clear. BTG2 has been found to be expressed at low levels in a variety of cancer cells, which has potential in the development and subsequent progression of tumors. Studies have shown that the expression level of BTG2 is low in breast cancer cell lines and is associated with tumor grade, size, metastasis, recurrence and breast cancer survival (25). Another study also reported low levels of BTG2 expression in renal cell carcinoma (33). Our study also showed a significant difference in the expression level of BTG2 gene in normal renal tissues and tumor tissues. However, it remains unclear whether m^6^A methylation modification influences BTG2 expression to alter epigenetic remodeling or contribute to malignant characteristics of RCC.

The findings of this study indicated that BTG2 suppresses RCC tumor growth. First, we found that the expression of BTG2 was repressed in RCC tumour tissues compared to normal tissues based on TCGA database. And patient outcomes were also adversely by BTG2 downregulation. Furthermore, we found that BTG2 inactivation might result from reduced m^6^A methylation modifications in the 5′UTR of mRNA. And Mettl3 regulates BTG2’s mRNA stability positively, thereby increasing the BTG2 m^6^A and expression levels. Nearly all stages in the lifecycle of RNA can be regulated by the m^6^A modification, including RNA processing, nuclear export, and translation (34, 35). The results of luciferase reporter system showed that m^6^A modification in 5′UTR region positively regulated m^6^A methylation level of BTG2 instead of CDS region. Moreover, we demonstrated that BTG2 mRNA stability was regulated by IGF2BP2 in RCC cells. It has also been shown that IGF2BPs could bind the GG(m^6^A)C sequence of mRNA to increase its stability and storage (26). As a result, our findings provide a fundamentally new understanding of the role of IGF2BP2 in RCC through its involvement in regulating the mRNA stability of BTG2.

Furthermore, CRIPSR/dCas13b-METTL3 was used specifically to methylate the m^6^A of BTG2 mRNA (28). It is also a newly developed method which can manipulate cell homeostasis by targeting specific methylation events in mRNA transcripts. The results showed that there was a 2-fold increase in m^6^A methylation level and a significant increase in BTG2 expression with this system. And dCas13b-M3 decreased RCC cells proliferation and migration and induced RCC cells apoptosis.

In summary, we demonstrate that m^6^A-mediated mRNA stability depends on methylation of 5′UTR regions in BTG2 mRNA to downregulate its expression. Our study further demonstrated that m^6^A regulates RCC cells growth and migration in RCC by regulating BTG2 expression. It is important to note that our results demonstrate that m^6^A methylation modification is instrumental in regulating the progression of RCC. Finally, METTL3/IGF2BP2/BTG2, a new regulatory complex, might provide novel insight into the pathogenesis and development of RCC.

## Methods

### Patients collection

We obtained renal cell carcinoma samples and adjacent nonmalignant renal tissues with informed consent from Shengli OilFiled Central Hospital’s (SOFCH) Urology Department. SOFCH’s Institutional Ethical Review Board approved this study in accordance with the Helsinki declaration. To isolate RNA from the samples, they were collected immediately after surgical removal and frozen in liquid nitrogen after rapid freezing.

### RNA-binding protein immunoprecipitation (RIP)

In accordance with the manufacturer’s instructions, RIP assays were performed with the Magna RIPTM RNA-Binding Protein Immunoprecipitation Kit (Millipore). A protease inhibitor cocktail and RNase inhibitor were included in the complete radioimmunoprecipitation assay buffer for lysing the cells. Protein A/G magnetic beads were pre-bound to antibodies (5 g) for 2 h before being incubated overnight with 100 L of cell lysate at 4°C. By incubating the beads in 400 μL of elution buffer for 2 h, eluting with ethanol, and dissolving in RNase-free water, the RNA was eluted from the beads. Real-time PCR was used to determine fragment enrichment.

### RT-qPCR, RIP-qPCR and MeRIP-qPCR

Following the instructions of Vazyme Biotech, Nanjing, China, total RNA was extracted from tissues and cell lines using the RNA-easy Isolation Reagent. Anti-m^6^A antibody-coupled beads were used to incubate fragmented RNA. IGF2BP2 was used to immunoprecipitate the m^6^A-containing RNA and then it was eluted from the beads. The RT-qPCR was performed with gene-specific primers on both input control and m^6^A-IP samples. A HiScript III RT SuperMix for qPCR was used to synthesize the cDNA (Vazyme Biotech, Nanjing, China). With universal SYBR Green qPCR Master Mix (Vazyme Biotech, Nanjing, China) and spectrophotometry (ABI Prism 7500TM instrument, Applied Biosystems), qRT-PCR was performed. The primers used in this study were listed in **Supplement Table S1**.

### Protein isolation and western blot

KeyGEN Bio TECH protein extraction kit (KGP1100) was used to extract protein from cells and separate it on 10% SDS-PAGE and transfer it to nitrocellulose membrane. According to previous instructions, blots were then immunostained with primary antibodies and secondary antibodies. The antibodies were as follows: BTG2 (1:1000; Abcam, United States) and GAPDH (1:10000; Proteintech, United States).

### Image quantification

Image density was quantified using ImageJ analysis software (NIH, Bethesda, MD, USA). And we are using a ratio to GAPDH.

### Expression plasmids, short interfering RNAs, and lentivirus transfection

Addgene provided the CRISPR dCas13b plasmids and Cas13b-gRNA plasmids. A number of designed gRNAs and the dCas13b-METTL3 vector have been constructed by Synbio Technologies. The overexpression plasmids were generated using the CDS of METTL3 or IGF2BP2 cloned into pcDNA3.1. The vector control used for analysis was pcDNA3.1.

### Cell culture and plasmid transfection

This study used cells from the RCC cell lines. Cell lines for this study was purchased from the American Type Culture Collection (ATCC, Manassas, VA, United States) and the National Infrastructure of Cell Line Resource, China. DMEM with 10% fetal bovine serum was routinely used for cell culture, supplemented with 5% CO2 in a 37°C environment (Invitrogen, Carlsbad, CA, United States). Lipo3000 (Invitrogen) was used for transfection of all plasmids as per the manufacturer’s instructions, and 1 μg of plasmids was used in each experiment.

### Luciferase reporter assay

In order to determine the effect of BTG2 expression, BTG2’s wild type or mutant 5’UTR was inserted at the end of the F-luc coding sequence. In both wild type and METTL3 overexpression cells, the pmirGLO-BTG2-5’UTR-WT and pmirGLO- BTG2-5’UTR-Mut were transfected for 24 hours. We analyzed firefly luciferase (F-luc) and renilla luciferase (R-luc) by using the Dual-Glo Luciferase Assay system (Promega) according to instructions. The activity of Renilla Luciferase (R-luc) was used to normalize the activity of Firefly Luciferase (F-luc) to evaluate reporter transcription. The experiments were repeated three times and the results were similar each time.

### mRNA stability

Cells transfected with different plasmids were stabilized with actinomycin D (Act-D, catalog #A9415, Sigma, U.S.A.) at 50 mg/ml during incubation. To conduct real-time PCR, RNA was isolated from the cells at the indicated times. BTG2 mRNA half-life was calculated by using ln2/slope and normalized using GAPDH.

### Cell proliferation and apoptosis assays

Detection of cell proliferation was carried out using the Cell Counting Kit 8 (CCK8) assay (Transgen, China). In 96-well plates, 3×10^3^ cells were seeded per well. A total of 24h, 48h, 72h, and 96h of cell culture was followed by 3h of incubation with CCK8 at 37°C. After that, 450 nm absorbance was measured using a microplate reader. Cells transfected with vectors were inoculated on a

12-well plate (2X10^5^ cells/well) with 70%–80% confluency. After 48 h, the cell was detected by the caspase-3/ELISA (enzyme-linked immunosorbent assay) (Hcusabio, China). The caspase-3 enzyme is a marker for inflammation and apoptosis signaling, as it can regulate the destruction of DNA or cytoskeletal proteins. Each test was performed at least three times.

### Wound healing migration assays

The wound healing migration experiments were carried out exactly as indicated. Cells were planted at a density of 5x10^5^ cells/well in a six-well chamber slide. A sterile 10 μL pipette tip was used to scratch the center of the slides. After 24 hours of incubation, photos were taken to assess the gap’s closure. The wound closure was measured using the following formulae:

### Statistical analyses

At least three independent experiments were used to gather data. Data are reported as mean + standard deviation. A two-tailed unpaired Student’s t-test was used between two groups, along with one-way or two-way ANOVA and Bonferroni testing for multiple comparisons. A two-sided test was used for all statistical analyses, using SPSS 16.0 for Windows. In order to be considered statistically significant, the p-value must be 0.05 or less. *p < 0.05, **p < 0.01; NS, no significant.

## Data availability statement

The datasets presented in this study can be found in online repositories. The names of the repository/repositories and accession number(s) can be found in the article/**Supplementary Material**.

## Ethics statement

The studies involving human participants were reviewed and approved by Shengli OilField Central Hospital's (SOFCH) Urology Department. The patients/participants provided their written informed consent to participate in this study.

## Author contributions

FQ, WL, and BT performed the experiments and data analysis. JZ and YM prepared diagrams and wrote the manuscript. FM, CC and BZ designed the project. JY and XL supervised the project and provided financial support. All authors contributed to the article and approved the submitted version.
