# Supplementary figures and images for: BTG2 suppresses renal cell carcinoma progression through N6-methyladenosine
Source: Front Oncol. 2022 Dec 14;12:1049928. doi: 10.3389/fonc.2022.1049928 (PMC9795213; doi:10.3389/fonc.2022.1049928)

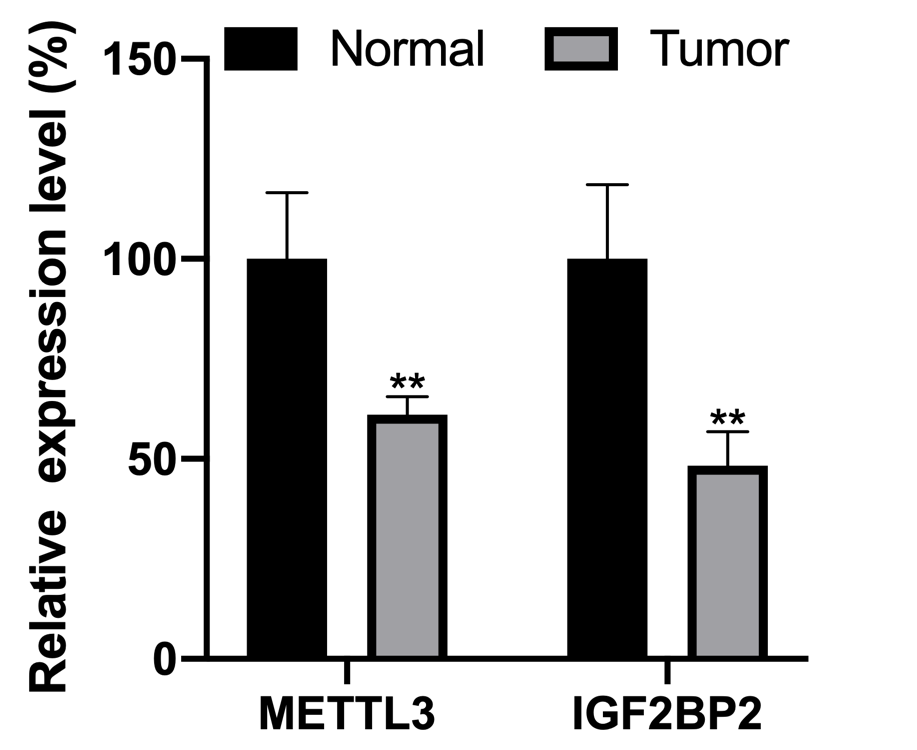

Supplement: Supplementary file 1 [file Image_1.tiff]

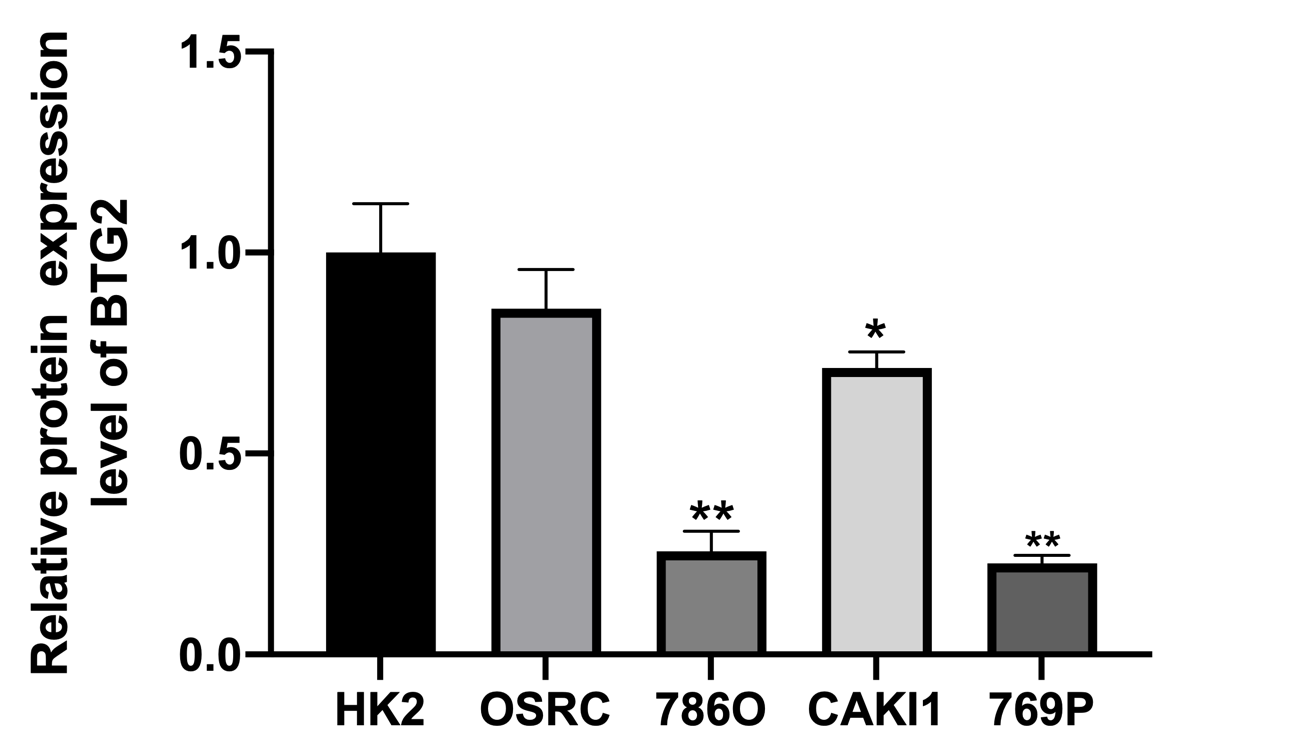

Supplement: Supplementary file 2 [file Image_2.tiff]

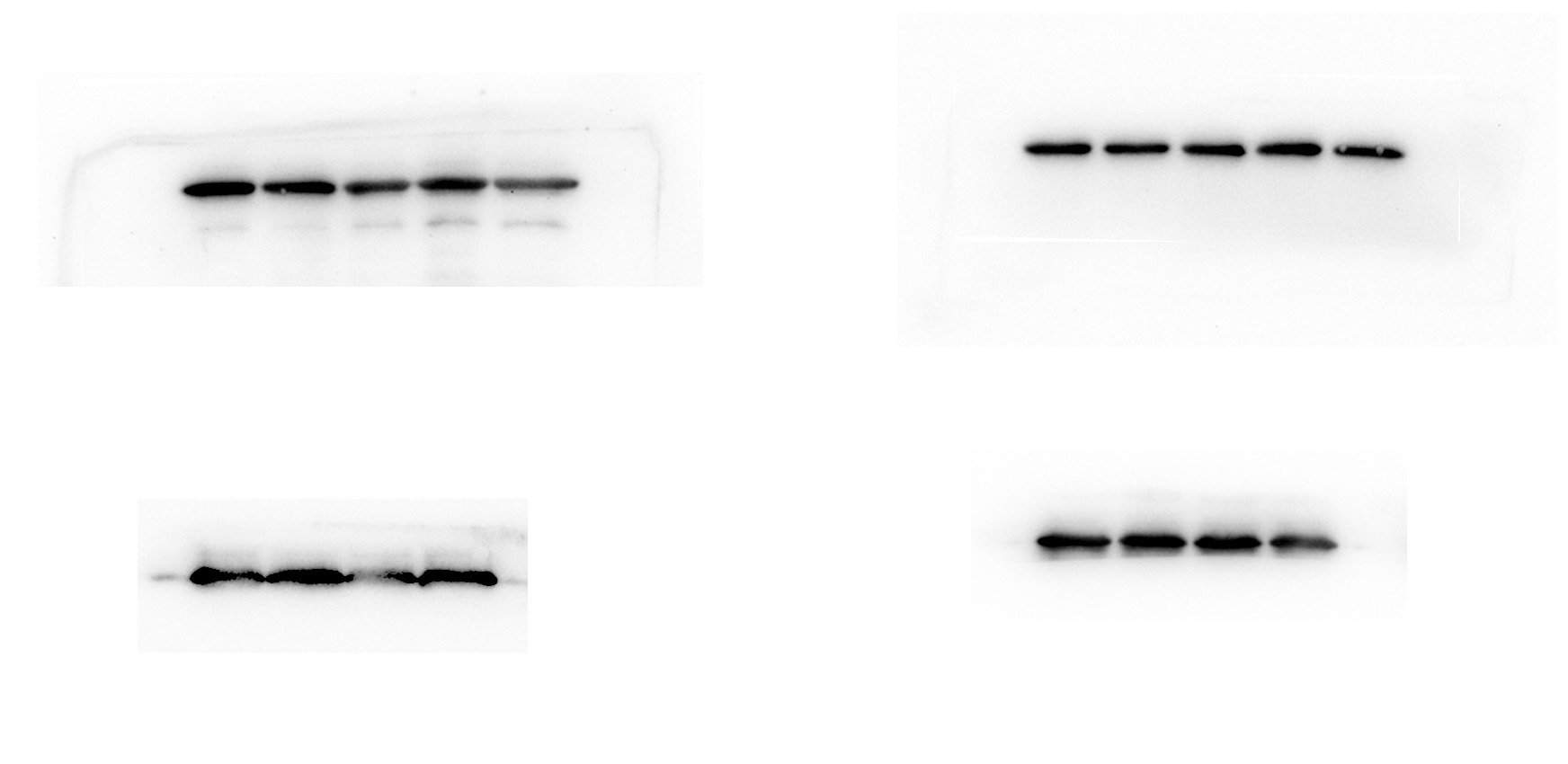

Supplement: Supplementary file 3 [file DataSheet_1.zip › Original data/Original image for checking.jpg]
